# Deep reinforcement learning for multi-class imbalanced training: applications in healthcare

**DOI:** 10.1007/s10994-023-06481-z

**Published:** 2023-11-28

**Authors:** Jenny Yang, Rasheed El-Bouri, Odhran O’Donoghue, Alexander S. Lachapelle, Andrew A. S. Soltan, David W. Eyre, Lei Lu, David A. Clifton

**Affiliations:** 1 Institute of Biomedical Engineering, Dept. Engineering Science, University of Oxford, Oxford, England; 2 Oxford Cancer & Haematology Centre, Oxford University Hospitals NHS Foundation Trust, Oxford, England; 3 RDM Division of Cardiovascular Medicine, University of Oxford, Oxford, England; 4 London Medical Imaging and AI Centre for Value Based Healthcare, Guy’s and St Thomas’ NHS Foundation Trust, London, England; 5 Big Data Institute, Nuffield Department of Population Health, University of Oxford, Oxford, England; 6 Oxford-Suzhou Centre for Advanced Research (OSCAR), Suzhou, China

**Keywords:** Reinforcement learning, Imbalanced learning, Imbalanced data

## Abstract

With the rapid growth of memory and computing power, datasets are becoming increasingly complex and imbalanced. This is especially severe in the context of clinical data, where there may be one rare event for many cases in the majority class. We introduce an imbalanced classification framework, based on reinforcement learning, for training extremely imbalanced data sets, and extend it for use in multi-class settings. We combine dueling and double deep Q-learning architectures, and formulate a custom reward function and episode-training procedure, specifically with the capability of handling multi-class imbalanced training. Using real-world clinical case studies, we demonstrate that our proposed framework outperforms current state-of-the-art imbalanced learning methods, achieving more fair and balanced classification, while also significantly improving the prediction of minority classes.

**Supplementary Information:**

The online version contains supplementary material available at 10.1007/s10994-023-06481-z.

## Introduction

In machine learning (ML), predictive modeling for classification tasks involves determining the class membership of a given observation. However, in many cases, the distribution of samples across different classes is skewed. This data imbalance presents a significant challenge in numerous real-world ML tasks, as imbalanced classification has been proven to lead to inferior predictive performance, particularly when it comes to the minority class (Haixiang et al., 2017; Kaur et al., 2019). Additionally, in cases where imbalance is more severe, conventional machine learning techniques often fall short since they were primarily developed under the assumption of equal class distribution during training (Ganganwar, 2012; Zong et al., 2013). While models can potentially learn from skewed distributions with an adequate amount of data, there are situations where classes exhibit similar conditional distributions, which has been shown to negatively affect model performance (Denil & Trappenberg, 2010). Thus, it is often necessary to employ specialized techniques in order to account for these data imbalances. This issue exists in many important domains, including fraud detection, anomaly detection, and disease diagnosis. In such cases, accurately classifying the minority class holds greater importance compared to the majority class. In this paper, we introduce a comprehensive framework that utilizes deep reinforcement learning to train algorithms on imbalanced datasets, encompassing both binary and multi-class scenarios. While the framework can be applied across disciplines, we focus on clinical applications for four reasons. First, the occurrence of disease in patients is generally less frequent compared to those without any disease, making it an ideal domain to investigate our framework. Second, because the issue of class imbalance is compounded by the multi-class nature of clinical problems, and by the dozens (sometimes hundreds) of possible diagnosis codes. Third, because of inter-disease heterogeneity. And finally, because a bias towards the majority class can have severe consequences in real-world settings, where patients belonging to a minority class could receive worse care.

### Related works

Two general categories of approaches have been introduced for overcoming data imbalance issues in machine learning: data-level approaches and algorithm-level approaches (Haixiang et al., 2017; Kaur et al., 2019; Tyagi & Mittal, 2020). Data-level solutions include re-sampling the training data to make it more balanced. There are two straightforward methods to achieve this: (1) adding instances from the underrepresented class (oversampling) or (2) removing instances from the overrepresented class (undersampling). However, both of these techniques have their drawbacks. Oversampling can increase the risk of overfitting as it introduces exact copies of the minority class, potentially leading to memorization rather than learning general rules for the minority class (He & Ma, 2013; Fernández et al., 2018). On the other hand, undersampling discards valuable data points, which can hinder the algorithm’s ability to learn the decision boundary between classes and lead to suboptimal performance (He & Ma, 2013; Fernández et al., 2018).

Another approach to oversampling involves generating synthetic samples for the underrepresented class. One commonly used method is the Synthetic Minority Oversampling Technique (SMOTE), which selects similar instances from the minority class and creates new instances by perturbing their attribute values within the range of the selected instances (Chawla et al., 2002). However, SMOTE does not take into account the majority class, which can result in ambiguous samples when there is significant overlap between the classes. Additionally, in clinical applications, evaluating the quality of synthetic data can be challenging, as there may not always be established clinical quality measures or evaluation metrics available (Chen et al., 2021). Moreover, in clinical variables where the data is categorical, perturbation may not be suitable or relevant, limiting the potential variability of the generated samples.

One commonly used approach at the algorithm level involves utilizing a penalized or cost-sensitive model. Cost-sensitive learning involves directly modifying an algorithm by assigning different weighted costs to account for the bias of each individual class. Since deep models are typically trained using backpropagation of a differentiable error term, errors from all classes are treated equally. This equal treatment can result in a skewed model that favors one class over others, especially in cases where the training data is imbalanced (Yang et al., 2023).

### Proposed method

To address the challenges associated with current methods, we propose the utilization of reinforcement learning (RL) for imbalanced classification tasks. RL offers a means of expressing errors through a non-differentiable signal that can be tailored to the specific situation. For instance, if it is crucial to detect a minority class, this can be represented through the reward function, which is typically not achievable through simple aggregation. Therefore, adopting a RL paradigm enables the learning of the minority class without compromising the learning of the majority class, implicitly. This is especially important in the context of the clinical tasks presented, where the goal is to train models capable of generalizing well across diverse patient outcomes, even when the distributions of these outcomes are imbalanced during model development.

RL (whereby an agent learns a task by trial and error, using a reward system) has demonstrated promising results for a wide variety of tasks, including classification. Namely, a branch of RL known as Q-learning, has been found to be successful for many applications, including those from a clinical domain. For example, the authors in Ling et al. (2017) developed a RL framework for patient diagnosis using clinical free text data. Here, a deep Q-network (DQN) was used, and preliminary results showed improvement over non-RL baselines. A DQN was also used for classification tasks in Martinez et al. (2018), where authors evaluated its efficacy for early classification using time-series data. Similarly, they found that RL could achieve effective performance over benchmarks.

With respect to imbalanced training, authors in Lin et al. (2020) showed that a DQN was effective for imbalanced classification. Using both image and text datasets, they demonstrated that their method achieved better balanced classification on imbalanced datasets than other imbalanced classification methods, such as over-/under-sampling, using cost-sensitive weights, and using decision-threshold adjustment. Although they demonstrated a strong RL-based classifier, they only evaluated their method on data imbalance ratios of up to 10%. However, in the case of clinical data (which we are focused on), there may be one rare event for hundreds or thousands of cases in the majority class. Furthermore, they only evaluated binary classification tasks; but often, a higher degree of granularity is required, as binning values to fewer (i.e. binary) classes may not be biologically relevant (especially when classes are categorical) and is heavily biased on the sample population (Miller et al., 2020). As it has previously been shown that a RL paradigm can be used for both image and text classification, the tasks presented here additionally demonstrate its effectiveness on tabular data.

## The Q-imb method

To extend the work in Lin et al. (2020), we formulated a framework that can be applied to multi-class classification problems, while being robust to extreme class imbalances ($\gg$ 90%). We trained dueling double deep Q-networks with a specialized reward function, for the purpose of mitigating data imbalances. We evaluated our framework on two independent, real-world, imbalanced-class clinical tasks - COVID-19 screening using anonymized electronic health record (EHR) data from hospital emergency rooms (binary prediction) and patient diagnosis in ICU wards using the eICU Collaborative Research Database (eICU-CRD) (multiclass prediction). Although we use clinical case studies, our methods can be adapted to many different classification tasks (including image recognition and natural language problems). Therefore, through our study, we hope to encourage and demonstrate the effectiveness of deep reinforcement learning on a wider range of prediction tasks, including those that are multi-class in nature, and may have extreme data imbalances.

To formulate classification as a RL task, we model our problem as a sequential decision-making task using a finite Markov Decision Process (MDP) framework, defined by a tuple of five variables (*s*, *a*, *r*, *p*, $\gamma$). During training, a batch of data is randomly shuffled and presented to the model in order, with each variable defined as follows:

- *s*: the state space of the process, where the features of each sample presented makes up the state
- *a*: the subsequent action that an agent takes, which is used to select a label for classification
- *r*: the expected reward for a given action, determined by the accuracy of classification
- *p*: the transition probability that results from an action, which in our case, is deterministic, as the agent moves from one state to the next according to the order of samples presented in the training data (i.e. the selection of an action, *a*, does not determine the next sample, *s*, presented to the agent)
- $\gamma$: the discount rate for future rewards

Given a *N*x*D* dataset, where *N* is number of samples, *D* is the feature dimensionality, and *K* is the number of classification categories; each sample *s*, has dimensionality *D*, and each action *a* is selected from one of *K* classes.

**Fig. 1:**
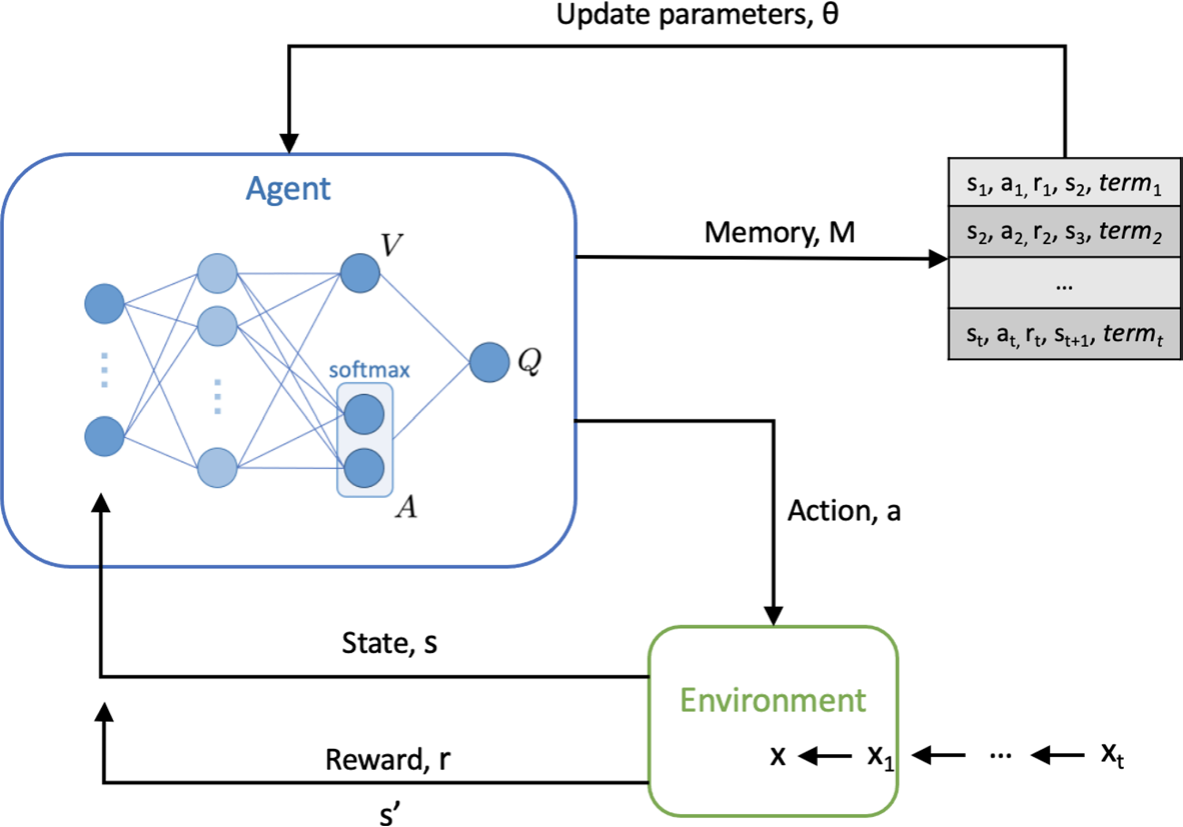
Overview of the reinforcement learning framework used. A dueling network architecture, with two streams to independently estimate the state-values (scalar) and advantages (vector) for each action, is shown

Our selection of an off-policy, model-free RL approach, specifically the Monte Carlo method, is intentional. An off-policy algorithm enables the presented samples to be independent and uncorrelated, and the model-free aspect means that we do not learn a transition function or trajectory, but rather focus on mapping states to appropriate actions for all states under consideration (Yang et al., 2023). Moreover, the use of temporal difference loss allows for more efficient estimation of the equivalent Monte Carlo return (Sutton, 1988), enabling us to treat each state independently in a feasible manner.

### Defining reward for multi-class imbalance

The reward, $r_t$, is the evaluation signal measuring the success of the agent’s selected action. A positive reward is given when the agent correctly classifies the sample, and a negative reward is given otherwise, thus allowing the agent to learn the optimal behavior for prediction. We let the reward for accurately/inaccurately labelling an instance of a particular class be inversely proportional to the relative presence of the class in the data. The absolute reward value of a sample from the minority class is thus higher than that in the majority class, making the model more sensitive to the minority class. With $l_k$ as the label of the sample, the reward function used is:

1 $$\begin{aligned} R(s_k, a_k, l_k)= & {} {\left\{ \begin{array}{ll} \lambda _k &{} \text { if }a_k = l_k\\ -\lambda _k &{} \text { if }a_k \ne l_k \end{array}\right. } \end{aligned}$$

2 $$\begin{aligned} \lambda _{\textrm{k}}= & {} \frac{\frac{1}{N_{k}}}{\sum _{i=1}^k |\frac{1}{N_{i}}|^2} \end{aligned}$$

$N_k$ represents the number of class instances in class *k* and $\lambda _k$ is a trade-off parameter used for adjusting the influence of the minority and majority classes. We found that our model achieved desirable performance when $\lambda _k$ is the sum of inverse squares of class frequencies, as shown in Eq. 2.

To balance immediate and future rewards, a discount factor, $\gamma \in [0,1)$, is typically used (used in Eq. 3 in the following section).

### Algorithmic adaptations for multi-class imbalance

To effectively perform RL under a multi-class imbalance setting, we propose the use of a dueling double deep Q-network (D-DDQN). We first begin by discussing the Q-value function. We then proceed to justify the use of dueling and double deep Q-learning components in the domain of class-imbalanced multi-class learning.

#### Policy iteration by Q-Learning

An optimal policy $\pi$* is a policy that maximizes $v^\pi$ (the value of a state-action combination), which can be found by iterating through a series of policies, $\{\pi \}_i^k$, where $\pi$*=$\pi ^k$. Using the Bellman equation, $v^\pi$ can be solved for through a system of linear equations and calculating a set of *Q*-values, where *Q* represents the action-value function:

3 $$\begin{aligned} Q_i^\pi (s_t,a_t)=r(s_t, a_t)+\gamma \sum _{s_{t+1}}p(s_{t+1}|s_t, a_t)v_i^\pi ( s_{t+1}), \end{aligned}$$

which gives successive policies:

4 $$\begin{aligned} \pi _{i+1}(a_t|s_t)=argmax_aQ_i^\pi (s_t, a_t) \end{aligned}$$

Here, the optimal policy is $\pi ^* = argmax_aQ^*$. Finally, to relate the state-action value function and *Q* function, the advantage function is used:

5 $$\begin{aligned} A^\pi (s_t,a_t)=Q^\pi (s_t,a_t)-V^\pi (s_t) \end{aligned}$$

#### Dueling Q-Network architecture

In the standard DQN setup, the output layer of the network corresponds to predicted Q-values for state-action pairs. In situations with a high number of possible state-action pairs such as multi-class prediction tasks, it becomes difficult to provide update information about the state because only one state-action pair in a state can be trained at a time. To alleviate this, the dueling DQN provides a method to train state representations independently of action representations. Furthermore, since instances only have one label, estimating the value of each state and reward for all actions in a given state isn’t realistic or appropriate, as instances only have one label. Thus, by using the advantage function in the dueling network, we estimate value for the only action the instance can be (i.e. its label).

For a DQN, the Q-network implemented is a single-stream neural network. This is a standard neural network with a continuous sequence of fully connected layers. The dueling Q-network instead implements a fully-connected neural network with two streams - one to estimate the value (scalar) and another to estimate the advantages of each action (vector). These two streams are combined to produce a single output, which is the *Q* function. This is known as a dueling network (Wang et al., 2016), and is shown in Fig. 1 (Supplementary Figure 1 in Section C of the Supplementary Material also shows a comparison of dueling and non-dueling architectures).

Based on the definition of the advantage function, *Q* is defined as:

6 $$\begin{aligned} Q_i^\pi (s_t,a_t;\;\theta _i,\alpha _i, \beta _i) =V_i^\pi (s_t;\;\theta _i,\beta _i)+ \bigg (A_i^\pi (s_t,a_t;\;\theta _i,\alpha _i)-softmax(A_i^\pi (s_t,a_{t+1};\;\theta _i,\alpha _i))\bigg ), \end{aligned}$$

where $\alpha$ and $\beta$ represent the parameters of the *A* and *V* streams of the network, respectively. The additional softmax module allows *Q* to recover *V* and *A* uniquely (it does not change the relative rank of *A* nor the Q-values, and thus preserves the $\epsilon$-greedy policy). A full explanation for adding this additional module can be found in Wang et al. (2016).

#### Double deep Q-learning

In each episode, the combinations of states, actions, and rewards at each step, $(s_t, a_t, r_t, s_{t+1})$, are stored in the agent’s working memory, *M*. A randomly sampled subset of these transitions *B*, are then used in the gradient descent step to learn the parameters of the Q-network. The Q-network is optimized using the mean-squared error loss function:

7 $$\begin{aligned} L(\theta _i)=\sum _{(s_t,a_t,r_t,s_{t+1})\in B}(y-Q(s_t,a_t; \theta _i))^2 \end{aligned}$$

As in traditional supervised learning, *y* and $Q(s,a; \theta _i)$ represent the target to be predicted and the prediction, respectively. To implement *y*, the formulation of a double deep Q-Network (DDQN) is used.

It has been shown that a standard DQN is more likely to give overoptimistic value estimates for actions (Thrun & Schwartz, 1993). A DQN uses the current Q-network to determine an action, as well as estimate its value. This increases the likelihood of selecting overestimated values (from the maximization step), making it harder to learn the optimal policy, as overestimations can occur even when action values are incorrect. This can become even more complex when there are a high number of possible state-action pairs, as seen in multi-class scenarios. Double deep Q-Learning was introduced as a method of reducing this overestimation (Van Hasselt et al., 2016). Unlike a DQN, a DDQN decouples the selection and evaluation steps, and instead, uses the current Q-network to select actions, and the target Q-network to estimate its value (Sui et al., 2018). Thus, a separate set of weights, $\theta '$, are used to provide an unbiased estimate of value.

We implement the DDQN algorithm using the following target function:

8 $$\begin{aligned} y_i= r_t+(1-term)\gamma Q(s_{t+1}, argmax_a Q(s_{t+1},a_{t+1};\theta _i); \theta _{i}') \end{aligned}$$

Since the selection of an action, *a*, does not directly determine the subsequent sample, *s*, presented to the agent, it is necessary to introduce an alternative dependency between *s* and *a*. In order to achieve this, we establish a connection by terminating a training episode when the agent incorrectly classifies the minority class, thereby discontinuing any further reward, *r*. Consequently, the variable *term* is assigned a value of 1 once the agent reaches its terminal state, and 0 otherwise, enabling the learning of a relationship between *s* and *a*. The terminal state is reached when the agent has either iterated through all samples in the training data (or a predetermined number of samples specified at the beginning of training) or when the agent misclassifies a sample from the minority class(es), effectively preventing any additional rewards. This training dependency, alongside the reward function, constitutes the overall environment reward procedure.

### Overall training procedure

During each episode, the agent selects an action using an $\epsilon$-greedy behavior policy. This randomly selects an action with probability $\epsilon$, or an action following the optimal Q-function, $argmax_aQ^*(s_t, a_t)$ with probability $1-\epsilon$. A reward is then given through the process summarized in Algorithm 1. The overall training procedure follows Algorithm 2, where the optimized Q-network is considered as the trained classifier. *T* is the number of samples specified at the beginning of training.

For the tasks presented here, each training period consisted of 120,000 steps, with a linearly attenuated exploration probability $\epsilon$ from 1 to 0.01 over the entire training process. For updating model weights, the Adaptive Moment Estimation (Adam) optimizer was used.
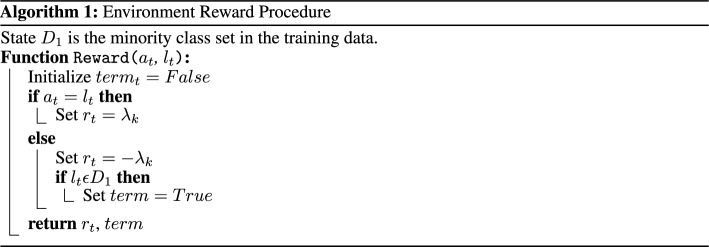

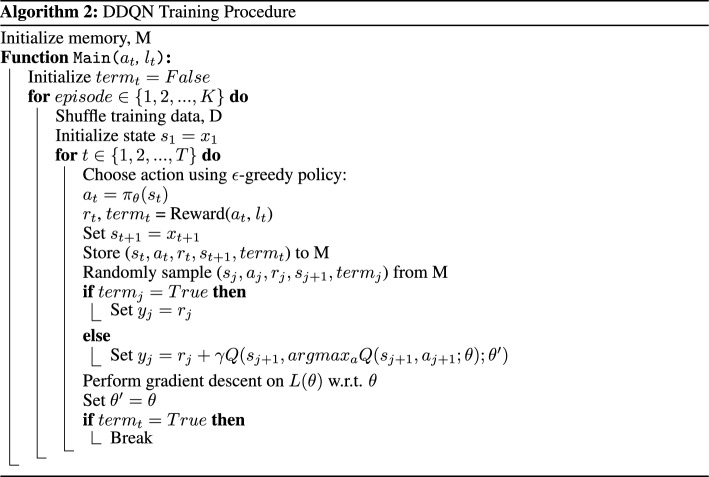


## Model comparators and evaluation metrics

We compare Q-imb against three baseline models - a fully-connected neural network and XGBoost (each with no added imbalanced learning strategy applied), as well as a DDQN and DQN (as introduced in Lin et al. (2020)) with no dueling component (implementation and training steps remain the same as Q-imb). We also present results for the neural network and XGBoost models with the addition of two commonly used, state-of-the-art imbalanced data-learning methods:

*SMOTE* SMOTE was applied to the training set using a minority oversampling strategy of 0.2 (i.e. the minority class was oversampled to have 20% of the number of samples in the majority class).

*Cost-Sensitive Learning* Different weighted costs were assigned to each class during training. The value of class weights chosen were inversely proportional to class frequencies in the training data.

We trained both a neural network and XGBoost model as-is, and additionally trained implementations that utilized SMOTE and cost-sensitive learning. Appropriate hyperparameter values for all models were determined through standard 5-fold cross-validation (CV), using the training set. For the DDQN without a dueling component, we use the same hyperparameter settings as Q-imb, to directly compare balanced classification performance of both methods. Details on network architecture and final hyperparameter values used for each model can be found in Section F of the Supplementary Material.

To evaluate the classification performance, we calculate the sensitivity, specificity, and the area under receiver operator characteristic curve (AUROC) across all test sets, alongside 95% confidence intervals (CIs) based on standard error. CIs for AUROC are calculated using Hanley and McNeil’s method.

Since our model’s objective is to train models effectively on imbalanced data, we assess the balanced classification performance using F-measure and G-mean metrics. The G-mean metric is calculated as the geometric mean of recall and specificity, while the F-measure represents the geometric mean of recall and precision (Lin et al., 2020). By utilizing geometric means, these metrics evaluate the sensitivity and specificity of the model, ensuring that both the true positive and true negative rates are adequately considered (Gu et al., 2009).

As used in Lin et al. (2020), we calculate F-measure and G-mean as follows:

9 $$\begin{aligned} F= & {} \sqrt{Sensitivity * Precision} \nonumber \\= & {} \sqrt{\frac{TP}{TP+FN} * \frac{TP}{TP+FP}} \end{aligned}$$

10 $$\begin{aligned} G= & {} \sqrt{Sensitivity * Specificity} \nonumber \\= & {} \sqrt{\frac{TP}{TP+FN} * \frac{TN}{TN+FP}} \end{aligned}$$

where TP is the number of true positives; FP is the number of false positives; TN is the number of true negatives; and FN is the number of false negatives.

## Prediction tasks and datasets

*COVID-19 Status Prediction* To show that Q-imb is effective for binary classification, we train models to predict the COVID-19 status for patients presenting to hospital emergency departments across four United Kingdom (UK) National Health Service (NHS) Trusts (Oxford University Hospitals NHS Foundation Trust [OUH], Portsmouth Hospitals University NHS Trust [PUH], University Hospitals Birmingham NHS Trust [UHB], Bedfordshire Hospitals NHS Foundations Trust [BH]), using anonymized EHR data (specifically, blood tests and vital sign features). We trained and optimized our model using 114,957 COVID-free patient presentations from OUH prior to the global COVID-19 outbreak, and 701 patient presentations during the first wave of the COVID-19 epidemic in the UK that had a positive PCR test for COVID-19 (ensuring that the label of COVID-19 status was correct during training). We then performed validation on a prospective OUH cohort, as well as external validation on three additional patient cohorts from PUH, UHB, and BH (totalling 72,223 admitted patients, including 4600 of which were COVID-19 positive). During training, we used a simulated disease prevalence of 5% (i.e. a data imbalance ratio of 1 positive COVID-19 case: 20 negative controls). This aligns with real COVID-19 prevalences at all four sites (during the dates of data extraction), which ranged between 4.27%$-$12.2%. Summary population characteristics for all patient cohorts and a full list of clinical predictors considered can be found in Supplementary Tables 1 and 2, respectively (Section D in the Supplementary Material). These are also presented alongside the full inclusion and exclusion criteria for patient cohorts, and data pre-processing steps.

*Patient Diagnosis* Further analysis was performed using the eICU Collaborative Research Database (eICU-CRD) (Pollard et al., 2018) which is publicly available through PhysioNet (Goldberger et al., 2000). Using this database, we tried to predict which of five acute events (cardiovascular, respiratory, gastrointestinal, systemic, renal) a patient is diagnosed with during their ICU stay. Through this task, we aimed to evaluate the utility of our model for a multi-class task, which is often necessary for many real-world applications. Prevalences across events ranged from 8.7%$-$33.6%, making this an appropriate task for investigating data imbalance effects. We trained our model using 18,076 samples and then tested it on 6026 held-out samples. Summary population characteristics for all patient cohorts, prevalence across diseases, and a full list of clinical predictors considered can be found in Supplementary Tables 4, 5, and 6, respectively (Section E in the Supplementary Material). These are also presented alongside the full inclusion and exclusion criteria for patient cohorts and, data pre-processing steps.

## Results

### COVID-19 diagnosis

Table 1 shows results for COVID-19 prediction, where we performed prospective validation and external validation across four NHS Trusts. Scores for F-measure and G-mean are presented alongside sensitivity, specificity, and AUROC (with 95% CIs). The results presented use an adjusted decision threshold, optimized to a sensitivity of 0.9. This threshold was chosen to ensure clinically acceptable performance in detecting positive COVID-19 cases, while also exceeding the sensitivities of current diagnostic testing methods (lateral flow device sensitivity was estimated to be around 57% (Soltan et al., 2022), real-time polymerase chain reaction has estimated sensitivities of approximately 80–90% (Williams et al., 2020; Miller et al., 2020)). In alignment with the NHS Trust policy’s green-amber-blue categorization system, we made the decision to employ binary classification (COVID-19 positive or negative) rather than utilizing probabilities. This categorization system assigns green to patients without any COVID-19 symptoms, amber to patients with symptoms that could potentially indicate COVID-19, and blue to patients with laboratory-confirmed COVID-19 infection (Miller et al., 2020; Soltan et al., 2022; Yang et al., 2023). By providing a classification outcome, our approach ensures consistency with the rapid triage process, allowing for patients to be efficiently sorted into either the green or amber pathway. Results achieved with no threshold adjustment (i.e., using the default threshold of 0.5) and when using a threshold of 0.85 can be found in Supplementary Tables 10 and 11, respectively (Section G of the Supplementary Material). As we are focused on evaluating balanced classification, we use red and blue to depict the best and second best scores, respectively, for F and G.

Results without any threshold adjustment (Supplementary Table 10), show that both baseline models performed poorly at predicting COVID-19 status (the minority class), achieving sensitivities below 0.5 on all test sets (mean sensitivities of 0.236 [CI range 0.071$-$0.388] and 0.340 [0.179$-$0.436] for neural network and XGBoost baseline models, respectively). The XGBoost model achieved slightly higher sensitivities than the neural network baseline, on all test sets. When SMOTE was applied to the training set, sensitivities slightly improved for both models (mean sensitivities of 0.463 [CI range 0.230$-$0.596] and 0.399 [0.205$-$0.528] for neural network and XGBoost models, respectively). When cost-sensitive learning is applied, the neural network model achieved much higher sensitivities than the baseline (mean sensitivity of 0.703 [CI range 0.539$-$0.785]); however, the XGBoost model only improved slightly with respect to its baseline comparator (mean sensitivity of 0.457 [CI range 0.303$-$0.578]). Compared to all baseline models and those additionally utilizing SMOTE and cost-sensitive weights, Q-imb achieved the highest sensitivities, without threshold adjustment, on all test sets (mean sensitivity of 0.806 [CI range 0.733$-$0.864]), while maintaining high specificity as well (mean specificity of 0.756 [0.669$-$0.871]). The RL models without a dueling component achieved high sensitivity (mean sensitivities of 0.838 [CI range 0.765$-$0.888] and 0.930 [0.902$-$0.991] for the DDQN and DQN, respectively); however, had much lower specificity (mean specificities of 0.357 [CI range 0.280$-$0.440] and 0.111 [0.068$-$0.138]), with the DDQN architecture slightly outperforming the DQN. Comparison of the output from Q-imb model to all other methods was found to be statistically significant (*p*<0.0001, by the Wilcoxon Signed Rank Test). Full numerical results can be found in Supplementary Table 10.

Although the models prior to threshold adjustment achieved poor performance on the minority class (except for Q-imb and the neural network with cost-sensitive weights), they still achieved reasonably high AUROC scores (>0.831, other than the RL methods without a dueling component, which achieved a slightly lower AUROC range of 0.659$-$0.762), suggesting that the models are able to distinguish between COVID-19 positive and negative classes. Thus, once threshold adjustment was applied, there was both higher and more balanced classification between COVID-19 positive and negative cases. The adjusted thresholds (listed in Supplementary Table 8) for Q-imb are also much closer to the default threshold (0.5) than the other methods, further demonstrating how threshold adjustment is not necessary even when the training data is heavily imbalanced (unlike the baseline models).

As our algorithm’s primary objective is to accurately screen for COVID-19, we assess the balanced classification performance of models that can reliably predict the COVID-19 status of individuals. Specifically, we focus on models that have been optimized to achieve a sensitivity of 0.9. As shown in Table 1, all models using this optimization achieved high sensitivities (>0.792).

In terms of balanced classification, Q-imb achieved the highest F and G scores for three test sets - OUH, UHB, and BH. The XGBoost models using SMOTE and cost-sensitive weights achieved the best F and G scores on the PUH dataset. Similar results were found for models optimized to sensitivities of 0.85, with Q-imb generally achieving the highest (or second highest) F and G scores, demonstrating model consistency. When no threshold adjustment was applied, Q-imb also achieved the highest (or second highest) G scores on all test sets; however, F scores were not as high compared to other models that had much lower sensitivity (<0.61) but very high specificity (>0.93), due to the nature of how F is calculated. The non-dueling DDQN and DQN models consistently achieved the lowest F and G scores, across all test sets (recall that it also achieved the lowest classification performance). Comparison of the output from Q-imb to all other methods was found to be statistically significant (p<0.0001, by the Wilcoxon Signed Rank Test).

All models trained achieved reasonably high AUROC scores across all test sets, comparable to those reported in previous studies, using the same patient cohorts (Miller et al., 2020; Soltan et al., 2022; Yang et al., 2022, 2023) (Supplementary Table 3), demonstrating that we have trained strong models to begin with. Thus, the results show that Q-imb is both a strong classifier, in addition to being able to account for large data imbalances.

Additional results evaluating the dueling component, as well as training time can be found in Supplementary Figures 2 and 3 (Section G in the Supplementary Material).

**Table 1:** Performance metrics for COVID-19 prediction

Model	F	G	AUROC	Sensitivity	Specificity
**OUH**					
Reinforcement learning (Q-imb)	**0**.**426**	**0**.**770**	0.861 (0.850$-$0.871)	0.838 (0.822$-$0.854)	0.707 (0.701$-$0.713)
Reinforcement learning (DDQN)	0.306	0.534	0.758 (0.745$-$0.771)	0.852 (0.836$-$0.867)	0.334 (0.328$-$0.341)
Reinforcement learning (DQN) Lin et al. (2020)	0.293	0.350	0.751 (0.739$-$0.764)	0.921 (0.909$-$0.933)	0.133 (0.128$-$0.138)
Neural network	0.388	0.715	0.877 (0.867$-$0.886)	0.899 (0.885$-$0.912)	0.568 (0.562$-$0.575)
Neural network + SMOTE	0.398	0.736	0.871 (0.861$-$0.881)	0.871 (0.856$-$0.885)	0.622 (0.615$-$0.628)
Neural network + cost-sensitive	0.400	0.737	0.872 (0.862$-$0.882)	0.881 (0.867$-$0.895)	0.616 (0.609$-$0.623)
XGBoost	0.399	0.734	0.877 (0.867$-$0.887)	0.889 (0.875$-$0.902)	0.607 (0.600$-$0.614)
XGBoost + SMOTE	*0.422*	*0.766*	0.876 (0.866$-$0.886)	0.846 (0.830$-$0.862)	0.694 (0.687$-$0.700)
XGBoost + cost-sensitive	0.399	0.739	0.869 (0.859$-$0.879)	0.857 (0.842$-$0.872)	0.637 (0.630$-$0.643)
**PUH**					
Reinforcement learning (Q-imb)	0.306	0.727	0.831 (0.819$-$0.842)	0.828 (0.812$-$0.845)	0.638 (0.633$-$0.643)
Reinforcement learning (DDQN)	0.248	0.606	0.762 (0.750$-$0.774)	0.804 (0.787$-$0.821)	0.457 (0.451$-$0.462)
Reinforcement learning (DQN) Lin et al. (2020)	0.223	0.324	0.732 (0.719$-$0.745)	0.915 (0.902$-$0.927)	0.115 (0.112$-$0.118)
Neural network	0.289	0.676	0.857 (0.847$-$0.868)	0.903 (0.890$-$0.916)	0.506 (0.501$-$0.511)
Neural network + SMOTE	0.309	0.728	0.856 (0.845$-$0.866)	0.859 (0.844$-$0.875)	0.617 (0.612$-$0.622)
Neural network + Cost-Sensitive	0.288	0.681	0.850 (0.839$-$0.861)	0.883 (0.869$-$0.897)	0.526 (0.521$-$0.531)
XGBoost	0.321	0.741	0.881 (0.871$-$0.891)	0.898 (0.884$-$0.911)	0.612 (0.607$-$0.617)
XGBoost + SMOTE	*0.325*	*0.750*	0.881 (0.871$-$0.890)	0.877 (0.863$-$0.892)	0.641 (0.636$-$0.646)
XGBoost + Cost-Sensitive	**0**.**336**	**0**.**766**	0.881 (0.871$-$0.891)	0.862 (0.847$-$0.877)	0.680 (0.675$-$0.684)
**UHB**					
Reinforcement learning (Q-imb)	**0**.**304**	**0**.**764**	0.837 (0.814$-$0.861)	0.815 (0.779$-$0.852)	0.717 (0.708$-$0.726)
Reinforcement learning (DDQN)	0.209	0.516	0.721 (0.694$-$0.749)	0.841 (0.806$-$0.875)	0.317 (0.308$-$0.326)
Reinforcement learning (DQN) Lin et al. (2020)	0.203	0.322	0.723 (0.695$-$0.750)	0.927 (0.903$-$0.951)	0.112 (0.106$-$0.118)
Neural Network	0.279	0.718	0.866 (0.844$-$0.888)	0.913 (0.887$-$0.940)	0.565 (0.555$-$0.574)
Neural network + SMOTE	0.290	0.746	0.850 (0.828$-$0.873)	0.845 (0.811$-$0.879)	0.658 (0.649$-$0.668)
Neural network + Cost-sensitive	0.284	0.733	0.861 (0.839$-$0.883)	0.879 (0.849$-$0.910)	0.611 (0.601$-$0.621)
XGBoost	0.287	0.740	0.861 (0.839$-$0.883)	0.872 (0.841$-$0.904)	0.627 (0.618$-$0.637)
XGBoost + SMOTE	*0.292*	*0.750*	0.853 (0.830$-$0.876)	0.827 (0.791$-$0.862)	0.680 (0.671$-$0.690)
XGBoost + cost-sensitive	0.289	0.746	0.851 (0.829$-$0.874)	0.838 (0.804$-$0.873)	0.663 (0.654$-$0.673)
**BH**					
Reinforcement learning (Q-imb)	**0**.**561**	**0**.**815**	0.867 (0.829$-$0.906)	0.806 (0.741$-$0.870)	0.825 (0.802$-$0.848)
Reinforcement learning (DDQN)	0.362	0.589	0.706 (0.656$-$0.756)	0.799 (0.733$-$0.864)	0.434 (0.403$-$0.464)
Reinforcement learning (DQN) Lin et al. (2020)	0.349	0.286	0.659 (0.608$-$0.710)	0.958 (0.926$-$0.991)	0.085 (0.068$-$0.102)
Neural network	0.525	0.802	0.885 (0.849$-$0.921)	0.868 (0.813$-$0.923)	0.741 (0.714$-$0.767)
Neural network + SMOTE	*0.540*	0.801	0.882 (0.845$-$0.919)	0.792 (0.725$-$0.858)	0.810 (0.786$-$0.834)
Neural network + Cost-sensitive	0.529	*0.804*	0.883 (0.847$-$0.920)	0.854 (0.797$-$0.912)	0.756 (0.730$-$0.782)
XGBoost	0.501	0.780	0.894 (0.859$-$0.929)	0.896 (0.846$-$0.946)	0.679 (0.650$-$0.707)
XGBoost + SMOTE	0.535	0.803	0.885 (0.849$-$0.921)	0.819 (0.757$-$0.882)	0.787 (0.762$-$0.812)
XGBoost + cost-sensitive	0.511	0.790	0.889 (0.854$-$0.925)	0.861 (0.805$-$0.918)	0.724 (0.697$-$0.751)

Results reported as F-measure, G-mean, AUROC, sensitivity, and specificity for OUH, PUH, UHB, and BH test sets; 95% confidence intervals (CIs) also shown. Bold and italics values denote best and second best scores, respectively, for F-measure and G-mean. Threshold adjustment applied to optimize models to sensitivities of 0.9

### Patient diagnosis prediction

For multiclass patient diagnosis, we calculated the individual sensitivities and G-means for all classes, using a “one-vs-all” method, for each method used, and present the mean sensitivities and G-means across all classes (Table 2).

**Table 2:** Performance metrics for multi-class diagnosis prediction

Model	G	Sensitivity
Reinforcement learning (Q-imb)	**0.834 (0.082)**	0.748 (0.126)
Reinforcement learning (DDQN)	0.776 (0.096)	0.671 (0.153)
Reinforcement learning (DQN) Lin et al. (2020)	0.777 (0.107)	0.672 (0.172)
Neural network	0.806 (0.105)	0.715 (0.183)
Neural network + SMOTE	0.804 (0.109)	0.714 (0.193)
Neural network + Cost-Sensitive	0.801 (0.103)	0.712 (0.190)
XGBoost	0.819 (0.106)	0.733 (0.181)
XGBoost + SMOTE	0.819 (0.107)	0.733 (0.184)
XGBoost + Cost-sensitive	*0.830 (0.092)*	0.744 (0.142)

Results reported as mean sensitivities and G values across all classes, shown alongside standard deviation

Bold and italics values denote best and second best scores, respectively, for G-mean

Here, all models achieved similar classification performances (mean sensitivity range of 0.712$-$0.748, other than the RL methods without a dueling component, which achieved a slightly lower mean sensitivities of 0.671 and 0.672 for the DDQN and the DQN, respectively) (Table 2). The highest mean sensitivity was achieved by the RL model (0.748 [SD 0.126]), followed closely by the XGBoost model with cost-sensitive weights (0.744 [0.142]). Comparison of the output from the RL model to all other methods was found to be statistically significant (p<0.0001, by the Wilcoxon Signed Rank Test).

There was a wide range in sensitivities for each acute event category across all models, varying from sensitivities of <0.5 to >0.9 (individual class performances can be found in Supplementary Table 12). Q-imb displayed the lowest variance (presented as standard deviation in Table 2) for sensitivities and G-means across all classes (SDs of 0.126 and 0.082 for sensitivity and G-mean, respectively), suggesting that it achieves the most balanced classification; and thus, is the least biased towards majority classes (individual class performances can be found in Supplementary Table 13). This is closely followed by the XGBoost model with cost-sensitive weights (SDs of 0.142 and 0.092 for sensitivity and G-mean, respectively). As seen in the previous task, the non-dueling DDQN and DQN models achieved the lowest mean sensitivity and mean G score, confirming how a policy that leads to good performance is harder to learn when state-action pairs are coupled. Q-imb also achieved either the highest (or second highest) sensitivities on the three classes with the lowest prevalences (acute gastrointestinal events, acute systemic events, acute renal events), across all methods. All methods performed poorly at predicting acute renal events, with only Q-imb and XGBoost with cost-sensitive weights achieving sensitivities >0.5 (0.545 [CI 0.508$-$0.58] and 0.515 [0.478$-$0.552] for the Q-imb and XGBoost models, respectively). Although performance on minority classes improved, the Q-imb model’s scores for the majority classes are slightly lower compared to other models. This was also the case for the XGBoost model with cost-sensitive weights, as the predictive performances of minority classes also improved at a slight cost of majority class performance. However, in both cases, the absolute rate of improvement was higher, leading to better overall performance.

Similar patterns were found with respect to G scores, as Q-imb achieved the highest mean G score (0.834 [SD 0.082]), followed by the XGBoost model with cost-sensitive weights (0.830 [0.092]), suggesting more balanced classification.

## Discussion and conclusion

As seen in many real-world ML studies, data imbalance poses a challenge, particularly with respect to correct classification of the minority class. This is especially evident in healthcare-related tasks, where the classification of the minority class is often more critical than the majority class. In this study, we used deep reinforcement learning, specifically in the context of imbalanced classification, and introduced a new formulation for multi-class settings. We evaluated our method against state-of-the-art imbalanced learning methods, using two challenging, real-world clinical case studies of COVID-19 diagnosis (binary) and general patient diagnosis (multi-class), with extreme data imbalances.

Experiments showed that our model achieved more balanced classification on imbalanced data than other imbalanced classification methods, significantly improving minority class sensitivity, while still achieving high majority-class performance. We also demonstrated that a dueling architecture was able to learn the state-value function, and therefore, the policy, more efficiently than a non-dueling comparator. Additionally, we showed that trained models were generalizable across four out-of-sample validation data sets (from four independent hospital trusts) with varying disease prevalences/imbalance ratios, which is a common disparity between hospitals and populations.

Although we were able to demonstrate that our method was effective for training imbalanced multi-class problems, it is still important to consider whether a class-specific model (i.e., using multiple one-vs-rest models) or a more general multi-class model is best suited for the task. Thus, future work should also consider the properties and nature of samples in minority classes, as this can give insight into the source of learning difficulties. For example, if there are different degrees of overlapping distributions between classes, it may be better to train multiple binary classifiers instead of a single multi-class one. Contrarily, for tasks where very large models need to be used, training multiple one-vs-rest models can overwhelm computing power; thus, using Q-imb in those scenarios would be beneficial.

With respect to the binary classification task, we used threshold adjustment to ensure models achieved high sensitivity for detecting COVID-19. Although threshold adjustment can be an effective strategy for achieving desirable detection rates, it is biased on the particular dataset the threshold is determined on. It has previously been shown that data can be biased towards site-specific factors (e.g. annotation, measuring devices, collection/processing methods, cohort distributions) Miller et al. (2020); Yang et al. (2023); thus, the threshold used at one site, may not be appropriate for use at a different site with varying distributions. This can make it difficult to perform external validation/translate tools to new, independent settings. However, as reinforcement learning can already achieve high sensitivities without having to use threshold adjustment, and additionally, learn an augmented representation of a task, it may have greater ability to generalize. This was demonstrated by the results, as Q-imb most consistently achieved the highest F and G scores across different optimization thresholds and across different test sites (whereas the neural network- and XGBoost-based methods showed greater variation across thresholds and test sites). Thus, choosing a decision threshold should be carefully considered, as it directly affects F and G metrics (through the shifting of sensitivity/specificity). Future experiments could consider using bespoke thresholds adapted to each independent dataset in order to improve classification performances on each set Yang et al. (2022).

When threshold adjustment is not applied, we found that Q-imb was the only method that achieved clinically-effective sensitivities for COVID-19 prediction. We found that both baseline methods (neural network and XGBoost without any imbalanced learning strategy applied), achieved poor sensitivities for COVID-19 prediction, with the XGBoost baseline achieving slightly better results. This was also found to be the case in the multiclass diagnosis task, as the baseline methods tended to have poorer performance on minority classes, with the XGBoost performing slightly better. This is expected, as most standard supervised learning models assume that classes are equally distributed Ganganwar (2012); Zong et al. (2013); and thus, skewed distributions can negatively affect the minority class Haixiang et al. (2017); Kaur et al. (2019). The XGBoost baseline model may have achieved slightly better sensitivity, as it is an ensemble method, which inherently combines the predictions of multiple models, improving the generalization error. However, sensitivity is still low, as ensemble methods still require a base classifier, which is usually a conventional machine learning model that typically isn’t suitable for imbalanced data Haixiang et al. (2017).

When SMOTE was added to training, both the neural network and the XGBoost model only improved slightly in detecting COVID-19 cases (mean sensitivities still <0.5). This may be because the added synthetic examples helped create larger (and thus, less specific) decision regions, making generalization easier. However, the improvement may have been minimal because new examples could have been generated from overlapping regions. This is especially relevant to health-related tasks, as clinical data is heterogeneous, and understanding how social, behavioral, and genetic factors collectively and independently impact outcomes is difficult Miller et al. (2020). Thus, it can be hard to confidently augment data, resulting in noisy regions. This is also reflected in the multiclass patient diagnosis task, where SMOTE did not impact XGBoost performance and decreased the performance of the neural network.

The addition of cost-sensitive weights improved sensitivities of both baseline models for COVID-19 prediction. This effect was especially noticeable with the neural network, as it achieved a much higher mean sensitivity compared to its baseline (mean sensitivity of 0.703 [CI range 0.539$-$0.785], from 0.236 [CI range 0.071$-$0.388]). This was also the case for the multiclass diagnosis task, as the sensitivity of minority classes also improved compared to respective baselines (however, at the cost of majority class prediction). This is expected, as cost-sensitive weights put more attention to samples from the minority class, allowing the backpropagation algorithm to assign weights to classification errors in proportion to the class imbalance.

In general, Q-imb achieved the highest (or second highest) G-mean scores across test sets, except for PUH when threshold adjustment was used. This may be related to the RL method also having poorer overall classification performance on PUH, possibly owing to site-specific differences (e.g. differences in protocols or methods used to collect and process data). Q-imb also achieved the highest (or second highest) F-scores when threshold adjustment was applied. When no threshold adjustment was applied, F scores were not as high compared to other models that had much lower sensitivity (<0.61), but very high specificity (>0.93). This is because these models generally had fewer false positives, which results in a higher F score (as shown in Eq. 12).

For the multiclass diagnosis task, we found that Q-imb achieved the strongest results in terms of classification performance and balanced performance (for both individual minority class scores and mean scores across all classes). However, all models achieved the lowest sensitivities for predicting acute renal events (which included electrolyte imbalances and renal failure). This may be because none of the markers typically used to diagnose these conditions, such a blood tests, urinalysis, urine output, swelling, or imaging studies, were included as input features to the model. The features available, such as blood pressure, heart rate, and oxygen saturation, primarily reflect the status of the cardiovascular and respiratory systems - as well as systemic function - which could explain why RL performed better in predicting these classes. We also note that the disease categories were neither mutually exclusive nor collectively exhaustive, and that a given patient might have several concurrent diagnosis across labels. Thus, future studies should consider adding more input features, including laboratory tests and imaging studies, and group diagnoses in a way that accounts for the fact that patient could be assigned to multiple labels. Additionally, multiclass tasks often require more data, and thus, there may not have been sufficient data to confidently differentiate between all classes, especially for this kind of challenging clinical task.

To conclude, as technological capabilities in memory and processing continue to advance, datasets are becoming much larger, complex, and imbalanced. In this paper, we discussed one domain - healthcare - where this is imbalance is especially severe; however, the framework introduced can be adapted to many other domains. As the method introduced is a total paradigm shift to previous imbalanced learning methods, and demonstrated with neural networks, these principles can be generalised to imbalanced image recognition problems, as well as NLP problems, which XGBoost (and other common baselines) are not appropriate for. Thus, as standard methods become inadequate for coping with such extreme levels of data imbalance, novel deep learning approaches will be key to propelling forward evidence-based AI.

### Supplementary Information

Below is the link to the electronic supplementary material.

Supplementary file 1 (pdf 432 KB)

## Data Availability

Data from OUH studied here are available from the Infections in Oxfordshire Research Database, subject to an application meeting the ethical and governance requirements of the Database. Data from UHB, PUH and BH are available on reasonable request to the respective trusts, subject to HRA requirements. The eICU Collaborative Research Database is available online.

## Code availability

Code can be found at https://github.com/yangjenny/ImbalancedLearningRL.
