# Supplementary material for: Deep reinforcement learning for multi-class imbalanced training: applications in healthcare
Source: Mach Learn. 2023 Nov 28;113(5):2655–74. doi: 10.1007/s10994-023-06481-z (PMC11065699; doi:10.1007/s10994-023-06481-z)
Supplement: Supplementary file 1 — (pdf 432 KB) [file 10994_2023_6481_MOESM1_ESM.pdf]

Supplementary Material

Algorithmic Fairness and Bias Mitigation for Clinical Machine Learning: A New Utility for Deep Reinforcement Learning

Jenny Yang, Rasheed El-Bouri, Odhran O'Donoghue, Alexander S. Lachapelle, Andrew A. S. Soltan, David W. Eyre, Lei Lu, & David A. Clifton, 2023

A Software Packages and Implementation

Models were implemented using Python (v3.6.9). Scikit Learn (v0.24.1) was used for standardization, median imputation, and calculating performance metrics. Imbalanced Learn (v0.7.0) was used to implement SMOTE. Performance metrics were calculated using Scikit Learn and manually programmed. XGBoost baseline models were implemented using the XGBoost library (v1.3.3). Neural network baseline models were implemented using Keras (v2.6.0). Reinforcement learning was set up using Tensorflow (v2.6.2). All models were run using an Intel Xeon E-2146G Processor (CPU: 6 cores, 4.50 GHz max frequency).

B Reinforcement Learning for Classification

Reinforcement learning (RL) has been linked to many real-world AI applications, with some of its most well-known successes stemming from game play and control challenges (e.g. AlphaGo [Silver et al., 2017], StarCraft [Vinyals et al., 2019], Atari games [Mnih et al., 2013], etc.). However, the core elements of RL have been shown to be successful on a wider range of tasks, including those that, on the surface, do not appear to have a particular "agent" interacting with an "environment" (which is typically regarded as the standard RL set-up [Sutton & Barto, 2018; Li, 2017]). Such problems include classification tasks, which have commonly been addressed using standard supervised learning algorithms, where an input through a model to predict a class label. RL, instead, uses an agent to interact with the input to determine which class it belongs to, and then receives an immediate reward from its environment based on that prediction. A positive reward is given to the agent when a label is correctly predicted, and a negative one is given otherwise. This feedback helps the agent learn the optimal "behavior" for classifying samples correctly, such that it accumulates the maximum rewards. This learned behavior is an augmented representation of the task, making it possible to learn beyond the immediate information encoded in the input (Wiering et al., 2011). To do this, an agent performs actions that set memory cells, which then can be used by the agent, together with the original input, to select actions and classify samples (Wiering et al., 2011; Lin et al., 2019).

C Model Architectures

C.1 Baseline Model Architectures

Neural Network: The rectified linear unit (ReLU) activation function was used for the hidden layers and the sigmoid activation function was used in the output layer. For updating model weights, the Adaptive Moment Estimation (Adam) optimizer was used during training.

XGBoost: XGBoost (Chen & Guestrin, 2016) is a popular ensemble model that has achieved state-of-the-art results on many machine learning challenges. Ensemble methods combine the predictions of multiple models, such that the generalization error is improves (i.e., contribution of individual error from any individual model is lessened). XGBoost in particular, utilizes a boosting technique, where trees are sequentially added and fit to correct for the prediction errors made by previous models. Default settings were used in all experiments.

C.2 Dueling Training Architecture

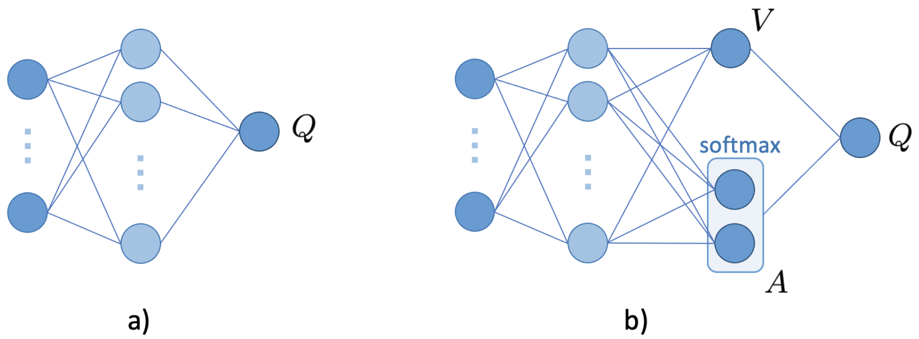

Figure 1: A typical single-stream Q-network is shown in a). A dueling architecture, with two streams to independently estimate the state-values (scalar) and advantages (vector) for each each action is shown in b) (this implements equation 9).

D COVID-19 Data and Preprocessing

D.1 Ethics statement

United Kingdom National Health Service (NHS) approval via the national oversight/regulatory body, the Health Research Authority (HRA), has been granted for development and validation of artificial intelligence models to detect Covid-19 using routinely collected hospital data (CURIAL; NHS HRA IRAS ID: 281832).

D.2 Data Inclusion and Exclusion

All data used is part of the NHS data for research and subject to data opt out (i.e. patients can apply to the NHS to stop their data from being used for research). Patients opting out of electronic health record (EHR) research were excluded.

The following inclusions and exlusions are reproduced from previous studies (Soltan et al., 2022, Yang et al., 2022a, Yang et al., 2022b).

Oxford University Hospitals NHS Foundation Trust (OUH): We included all patients attending acute and emergency care settings at OUH who received routine blood tests on arrival, considering presentations before December 1, 2019, and thus before the pandemic, as the COVID-19-negative (control) cohort. We considered presentations during the 'first wave' of the UK COVID-19 pandemic (December 1, 2019 to June 30, 2020) with PCR confirmed SARS-CoV-2 infection as the COVID-19-positive (cases) cohort. We excluded patients who did not receive laboratory blood tests or were younger than 18 years of age. Due to incomplete penetrance of testing during the first wave of the pandemic, and imperfect sensitivity of the PCR test, there is uncertainty in the viral status of patients presenting during the pandemic who were untested or tested negative. We therefore selected a pre-pandemic control cohort during training to ensure absence of disease in patients labelled as COVID-19-negative. Clinical features extracted for each presentation included first-performed blood tests, blood gases, vital signs measurements and PCR testing for SARS-CoV-2 (Abbott Architect [Abbott, Maidenhead, UK], TaqPath [Thermo Fisher Scientific, Massachusetts, USA] and Public Health England-designed RNA-dependent RNA polymerase assays).

Portsmouth Hospitals NHS Foundation Trust (PUH): PUH considered all patients admitted to the Queen Alexandria Hospital, serving a population of 675,000 and offering tertiary referral services to the surrounding region, between March 1, 2020 and February 28, 2021. Confirmatory COVID-19 testing was by laboratory SARS-CoV2 RT-PCR assay, considering any positive PCR result within 48hrs of admission as a true positive.

University Hospitals Birmingham NHS Foundation Trust (UHB): UHB considered all patients admitted to The Queen Elizabeth Hospital, Birmingham, between December 01, 2019 and October 29, 2020. The Queen Elizabeth Hospital is a large tertiary referral unit within the UHB group which

provides healthcare services for a population of 2.2 million across the West Midlands. Confirmatory COVID-19 testing was performed by laboratory SARS-CoV-2 RT-PCR assay.

Bedfordshire NHS Foundation Trust (BH): BH considered all patients admitted to Bedford Hospital between January 1, 2021 and March 31, 2021. BH provides healthcare services for a population of around 620,000 in Bedfordshire. Confirmatory COVID-19 testing was performed on the day of admission by point-of-care PCR based nucleic acid testing [SAMBA-II & Panther Fusion System, Diagnostics in the Real World, UK, and Hologic, USA].

Table 1: Summary population characteristics for OUH training cohorts, prospective validation cohort of patients attending OUH, independent validation cohorts of patients admitted to three independent NHS Trusts. *indicates merging for statistical disclosure control.

	OUH (pre-pandemic & wave 1 cases, to 30/06/2020)		OUH	PUH	UHB	BH
Cohort	Pre-pandemic cohort	COVID-19-cases cohort	01/10/2020-06/03/2021	01/03/2020-28/02/2021	01/12/2019-29/10/2020	01/01/2021-31/03/2021
**n, patients**	114,957	701	22,857	37,896	10,293	1177
**n, COVID positive**	0	701	2,012 (8.80%)	2,005 (5.29%)	439 (4.27%)	144 (12.2%)
**Sex:**						
- Male (%)	53370 (46.43)	376 (53.64)	11409 (49.91)	20839 (54.99)	4831 (46.93)	627 (53.27)
- Female (%)	61587 (53.57)	325 (46.36)	11448 (50.09)	17054 (45.0)	5462 (53.07)	549 (46.64)
Age, yr (IQR)	60 (38-76)	72 (55-82)	67 (49-80)	69 (48-82)	63 (42-79)	68.0 (48-82)
**Ethnicity:**						
-White (%)	93921 (81.7)	480 (68.47)	17387 (76.07)	28704 (75.74)	6848 (66.53)	1024 (87.0)
-Not Stated (%)	13602 (11.83)	128 (18.26)	4127 (18.06)	8389 (22.14)	1061 (10.31)	$\leq$10
-South Asian (%)	2754 (2.4)	22 (3.14)	441 (1.93)	170 (0.45)	1357 (13.18)	71 (6.03)
-Chinese (%)	284 (0.25)	*	51 (0.22)	42 (0.11)	41 (0.4)	$\leq$10
-Black (%)	1418 (1.23)	25 (3.57)	279 (1.22)	187 (0.49)	484 (4.7)	36 (3.06)
-Other (%)	1840 (1.6)	34 (4.85)*	410 (1.79)	269 (0.71)	333 (3.24)	29 (2.46)
-Mixed (%)	1138 (0.99)	12 (1.71)	162 (0.71)	135 (0.36)	169 (1.64)	13 (1.1)

Table 2: Clinical predictors considered.

**Category**	**Features**
Vital Signs	Heart rate, respiratory rate, systolic blood pressure, diastolic blood pressure, temperature
Full Blood Count	Haemoglobin, haematocrit, mean cell volume, white cell count, neutrophil count, lymphocyte count, monocyte count, eosinophil count, basophil count, platelets
Liver Function Tests & C-reactive protein	Albumin, alkaline phosphatase, alanine aminotransferase, bilirubin, C-reactive protein
Urea & Electrolytes	Sodium, potassium, creatinine, urea, estimated glomerular filtration rate

D.3 Preprocessing

We used electronic health record (EHR) data with linked, deidentified demographic information for all patients presenting to emergency departments. To better compare our results to previously published studies using the same datasets (Soltan et al., 2022, Yang et al., 2022a, Yang et al., 2022b), we used the same focused subset of routinely collected clinical features (including blood tests and vital signs) and patient cohorts.

The OUH training set consisted of COVID-free cases prior to the outbreak, so we matched every COVID-positive case to twenty COVID-free presentations based on age, representing a simulated prevalence of 5%. Consistent with previous studies, we also used population median imputation to replace any missing values. We then standardized all features in our data to have a mean of 0 and a standard deviation of 1.

A training set was used for model development, hyperparameter selection, and training; a validation set was used for threshold-adjustment; and after successful development and training, held-out test sets were then used to evaluate the performance of the final model. Hyperparameters and thresholds values used in the final models can be found in Supplementary Tables 7, 8, and 9 (Section F in the Supplementary Material).

Table 3: Previously published COVID-19 status prediction results. using same datasets and patient cohorts. Sensitivity, specificity, and AUROC shown, alongside 95% confidence intervals, unless otherwise specified.

Test Set	Sensitivity	Specificity	AUROC
**Soltan et al., 2022.**			
Method: XGBoost + SMOTE + Threshold Adjustment (0.9)			
OUH	0.857 (SD 0.009)	0.686 (SD 0.022)	0.878 (SD 0.001)
PUH	0.841 (0.825-0.857)	0.713 (0.709 -0.718)	0.872 (0.863 -0.882)
UHB	0.788 (0.748-0.824)	0.747 (0.738 -0.755)	0.858 (0.838 -0.878)
BH	0.743 (0.666-0.807)	0.848 (0.825 0. 869)	0.881 (0.851- 0.912)
**Yang et al., 2022.**			
Method: Neural Network + SMOTE + ENN + Threshold Adjustment (0.85)			
OUH	0.844 (0.828-0.860)	0.710 (0.704-0.717)	0.777 (0.765-0.789)
PUH	0.857 (0.842-0.873)	0.672 (0.667-0.677)	0.765 (0.752-0.777)
UHB	0.847 (0.814-0.881)	0.716 (0.708-0.725)	0.782 (0.756-0.808)
BH	0.847 (0.789-0.906)	0.822 (0.799-0.845)	0.835 (0.793-0.876)
**Yang et al., 2022.**			
Method: Neural Network + Threshold Adjustment (0.85)			
OUH	0.762 (0.744-0.781)	0.844 (0.839-0.849)	0.878 (0.868-0.888)
PUH	0.633 (0.585-0.681)	0.903 (0.897-0.910)	0.861 (0.837-0.885)
UHB	0.714 (0.621-0.807)	0.854 (0.839-0.870)	0.878 (0.832-0.924)
BH	0.724 (0.561-0.887)	0.908 (0.869-0.948)	0.880 (0.798-0.963)

E Multiclass Patient Diagnosis Data and Preprocessing

E.1 Ethics statement

The eICU Collaborative Research Database (eICU-CRD) is a publicly-available, anonymized database with pre-existing institutional review board (IRB) approval. The database is released under the Health Insurance Portability and Accountability Act (HIPAA) safe harbor provision. The re-identification risk was certified as meeting safe harbor standards by Privacert (Cambridge, MA) (HIPAA Certification no. 1031219-2).

E.2 Data Inclusion and Exclusion

In terms of clinical applications of AI, patient diagnosis as been a popular problem to address (Sheikhalishahi et al., 2021; Lipton et al., 2015; Razavian et al., 2016), as it can directly influence clinical decision-making, resource allocation, and healthcare costs.

Here, the task was to predict which acute condition might be developed by a patient during the course of an ICU stay, as defined through ICD-9 codes. A similar task that included both acute and chronic conditions was previously investigated using the eICU-CRD dataset by grouping 767 ICD-9 codes into 25 overarching diagnoses, and then predicting these using a BiLSTM model (Sheikhalishahi et al., 2021). Using similar inclusion and exclusion criteria, we selected adult patients (age > 18) with a minimum of 15 ICU records, and grouped these records into 1 hour windows. Our clinical team reviewed the list of 25 diagnoses, removed 13 diagnoses considered chronic, non-acute, or poorly defined, and grouped the remaining 12 diagnoses into their relevant system and clinical specialties. This resulted in five labels: acute cardiovascular event, acute respiratory event, acute gastrointestional event, acute systemic event, and acute renal event. This grouping was selected to reflect clinic reality, where an emergency physician might consult with a system specialist to rule out a severe condition before admission to ICU, and to account for the relatedness of diagnoses within a system. For example, pneumonia is a leading cause of respiratory failure, and combining both diagnoses into a single "acute respiratory event" category reflects the systemic nature of the disease. We removed any samples that did not have a differentiable ICD9 code, or did not belong to any of the curated groups, resulting in 24,102 samples for training and testing.

Table 4: Summary population characteristics for eICU-CRD cohort.

Characteristic	eICU-CRD Cohort
Sex:<br>Female (%)	10842 (45.0)
Male (%)	13260 (55.0)
Age (IQR)	65 (54-76)
Ethnicity:<br>Unknown (%)	1462 (6.1)
Asian (%)	479 (2.0)
African American (%)	2557 (10.6)
Caucasian (%)	18440 (76.5)
Hispanic (%)	1002 (4.2)
Native American (%)	162 (0.67)

Table 5: Acute event groups and respective prevalences.

Label	Events	Prevalence
Acute cardiovascular event	Acute myocardial infarction, acute cerebrovascular disease	0.288
Acute respiratory event	Respiratory failure, insufficiency, arrest, pneumonia, pleurisy, pneumothorax, pulmonary collapse, other upper respiratory disease, other lower respiratory disease	0.336
Acute gastrointestinal event	Gastrointestinal hemorrhage	0.087
Acute systemic event	Septicemia, shock	0.174
Acute renal event	Renal failure, fluid and electrolyte disorder	0.113

Table 6: Clinical predictors considered for predicting patient discharge status and patient diagnosis.

Category	Features
Demographic features	Gender, age, ethnicity, height, weight
Measurements at hospital admission	Non-invasive systolic blood pressure, non-invasive diastolic blood pressure, non-invasive mean arterial pressure, heart rate, Supporting oxygen used at admission, blood oxygen saturation, Glasgow coma score, diagnosis at admission
Measurements at ICU admission	Glucose

E.3 Preprocessing

Further preprocessing was performed to remove samples with any missing values, one-hot encode categorical features, and standardize all continuous features to have a mean of 0 and a standard deviation of 1.

We used a 75:25 training and test ratio, resulting in 18,076 training and 6,026 test samples, respectively. As before, the training set was used for model development, hyperparameter selection, and training; and after successful development and training, the held-out test set was used to evaluate the performance of the final model. It should be noted that, as this is a multiclass task, standard threshold adjustment cannot be used, and thus, we did not split the data to include an additional validation set.

F Hyperparameter Values and Decision Thresholds

F.1 COVID-19 Diagnosis

Table 7: Final Hyperparameter Values Used in Reinforcement Learning, Neural Network, and XGBoost-Based Models in COVID-19 Prediction Task

**Reinforcement Learning**	**Neural Network**	**XGBoost**
Dropout = 0.3<br>Learning rate = 0.0004<br>Neurons = 100<br>Discount Factor = 0.1<br>Exploration Factor = [0.01, 1]	Dropout = 0.3<br>Learning rate = 0.1<br>Neurons = 10	Depth = 3<br>N estimators = 100<br>Learning rate = 0.1

We implemented early stopping, which monitored validation performance, optimizing training for a sensitivity of >0.85 and specificity of >0.75. These thresholds were set to ensure that the model would be able to detect positive COVID-19 cases.

Table 8: Adjusted Threshold Values Used in Reinforcement Learning, Neural Network, and XGBoost Models, for COVID-19 status prediction.

Model	Threshold	
	0.85	0.9
Reinforcement Learning (Q-imb)	0.5050	0.4970
Reinforcement Learning (DDQN)	0.5070	0.5010
XGBoost	0.0060	0.0020
XGBoost + SMOTE	0.0120	0.0060
XGBoost + Cost-Sensitive	0.0120	0.0060
NN	0.0341	0.0140
NN + Cost-Senstive	0.4148	0.2645
NN + SMOTE	0.0862	0.0641

F.2 Multiclass Patient Diagnosis

Table 9: Final Hyperparameter Values Used in Reinforcement Learning, Neural Network, and XGBoost-Based Models in ICU Diagnosis Prediction Task

**Reinforcement Learning**	**Neural Network**	**XGBoost**
Dropout = 0.3<br>Learning rate = 0.0001<br>Neurons = 3000<br>Discount Factor = 0.1<br>Exploration Factor = [0.01, 1]	Dropout = 0.3<br>Learning rate = 0.01<br>Neurons = 200	Depth = 3<br>N estimators = 100<br>Learning rate = 0.1

G Additional Results

G.1 DDQN and Dueling DDQN Comparison

The dueling DDQN consistently outperforms the DDQN, across all four test sets. This can also be seen in the training curves, as the dueling DDQN appears to be able to learn a better policy.

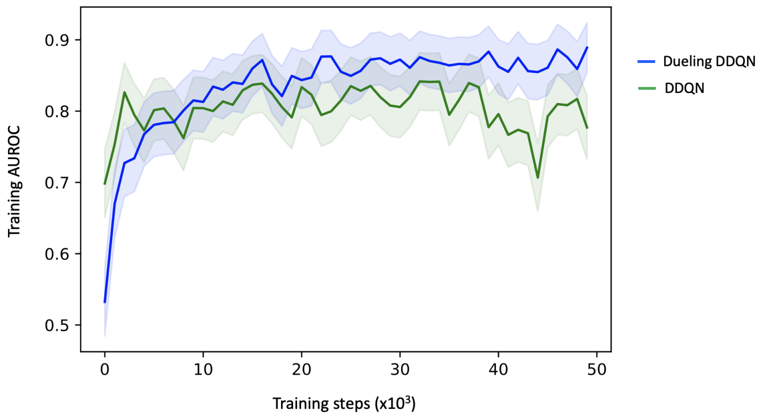

Figure 2: AUROC scores during training, comparing DDQN and Dueling DDQN models. Curves are shown for the COVID-19 prediction task.

G.2 COVID-19 Diagnosis

Table 10: Performance metrics for COVID-19 prediction. Results reported as F-measure, G-mean, sensitivity, specificity, and AUROC for OUH, PUH, UHB, and BH test sets; 95% confidence intervals (CIs) also shown. Red and blue values denote best and second best scores, respectively for F-measure and G-mean. No threshold adjustment was applied (i.e. default threshold of 0.5 used for prediction)

Model	F	G	Sensitivity	Specificity	AUROC
**OUH**					
Reinforcement Learning (Q-imb)	0.441	**0.782**	0.819 (0.802-0.835)	0.746 (0.740-0.752)	0.861 (0.850-0.871)
Reinforcement Learning (DDQN)	0.304	0.515	0.863 (0.848-0.878)	0.308 (0.301-0.314)	0.758 (0.745-0.771)
Neural Network	0.470	0.502	0.253 (0.234-0.272)	0.996 (0.996-0.997)	0.877 (0.867-0.886)
Neural Network + SMOTE	0.593	0.706	0.510 (0.489-0.532)	0.978 (0.976-0.980)	0.871 (0.861-0.881)
Neural Network + Cost-Sensitive	0.507	**0.795**	0.723 (0.703-0.742)	0.874 (0.869-0.878)	0.872 (0.862-0.882)
XGBoost	0.577	0.623	0.391 (0.369-0.412)	0.994 (0.992-0.995)	0.877 (0.867-0.887)
XGBoost + SMOTE	**0.595**	0.657	0.435 (0.414-0.457)	0.990 (0.989-0.992)	0.876 (0.866-0.886)
XGBoost + Cost-Sensitive	**0.584**	0.685	0.478 (0.456-0.499)	0.982 (0.980-0.983)	0.869 (0.859-0.879)
**PUH**					
Reinforcement Learning (Q-imb)	0.316	**0.741**	0.814 (0.797-0.831)	0.674 (0.669-0.678)	0.831 (0.819-0.842)
Reinforcement Learning (DDQN)	0.245	0.594	0.810 (0.793-0.827)	0.435 (0.430-0.440)	0.762 (0.750-0.774)
Neural Network	0.47	0.601	0.367 (0.345-0.388)	0.987 (0.985-0.988)	0.857 (0.847-0.868)
Neural Network + SMOTE	0.452	0.735	0.574 (0.552-0.596)	0.942 (0.940-0.944)	0.856 (0.845-0.866)
Neural Network + Cost-Sensitive	0.356	**0.775**	0.767 (0.748-0.785)	0.784 (0.780-0.789)	0.850 (0.839-0.861)
XGBoost	**0.504**	0.639	0.414 (0.393-0.436)	0.985 (0.984-0.987)	0.881 (0.871-0.891)
XGBoost + SMOTE	**0.521**	0.702	0.506 (0.484-0.528)	0.976 (0.974-0.977)	0.881 (0.871-0.890)
XGBoost + Cost-Sensitive	**0.521**	0.734	0.557 (0.535-0.578)	0.967 (0.966-0.969)	0.881 (0.871-0.891)
**UHB**					
Reinforcement Learning (Q-imb)	0.315	**0.772**	0.793 (0.755-0.831)	0.753 (0.744-0.761)	0.837 (0.814-0.861)
Reinforcement Learning (DDQN)	0.206	0.494	0.845 (0.811-0.879)	0.289 (0.280-0.298)	0.721 (0.694-0.749)
Neural Network	0.351	0.444	0.198 (0.161-0.235)	0.995 (0.993-0.996)	0.866 (0.844-0.888)
Neural Network + SMOTE	**0.418**	0.667	0.460 (0.414-0.507)	0.966 (0.963-0.970)	0.850 (0.828-0.873)
Neural Network + Cost-Sensitive	0.364	**0.780**	0.704 (0.661-0.747)	0.864 (0.858-0.871)	0.861 (0.839-0.883)
XGBoost	0.417	0.548	0.303 (0.260-0.346)	0.990 (0.988-0.992)	0.861 (0.839-0.883)
XGBoost + SMOTE	**0.431**	0.608	0.376 (0.331-0.421)	0.983 (0.980-0.985)	0.853 (0.830-0.876)
XGBoost + Cost-Sensitive	0.410	0.633	0.412 (0.366-0.458)	0.973 (0.970-0.976)	0.851 (0.829-0.874)
**BH**					
Reinforcement Learning (Q-imb)	**0.582**	**0.823**	0.799 (0.733-0.864)	0.849 (0.827-0.871)	0.867 (0.829-0.906)
Reinforcement Learning (DDQN)	0.364	0.573	0.826 (0.765-0.888)	0.397 (0.367-0.427)	0.706 (0.656-0.756)
Neural Network	0.306	0.353	0.125 (0.071-0.179)	0.994 (0.990-0.999)	0.885 (0.849-0.921)
Neural Network + SMOTE	0.481	0.549	0.306 (0.230-0.381)	0.986 (0.979-0.993)	0.882 (0.845-0.919)
Neural Network + Cost-Sensitive	**0.586**	**0.759**	0.618 (0.539-0.697)	0.931 (0.916-0.947)	0.883 (0.847-0.920)
XGBoost	0.457	0.498	0.250 (0.179-0.321)	0.993 (0.988-0.998)	0.894 (0.859-0.929)
XGBoost + SMOTE	0.491	0.526	0.278 (0.205-0.351)	0.994 (0.990-0.999)	0.885 (0.849-0.921)
XGBoost + Cost-Sensitive	0.556	0.614	0.382 (0.303-0.461)	0.987 (0.981-0.994)	0.889 (0.854-0.925)

Table 11: Performance metrics for COVID-19 prediction. Results reported as F-measure, G-mean, sensitivity, specificity, and AUROC for OUH, PUH, UHB, and BH test sets; 95% confidence intervals (CIs) also shown. Red and blue values denote best and second best scores, respectively for F-measure and G-mean. Threshold adjustment applied to optimize models to sensitivities of 0.85.

Model	F	G	Sensitivity	Specificity	AUROC
**OUH**					
Reinforcement Learning (Q-imb)	**0.462**	**0.792**	0.791 (0.773-0.809)	0.793 (0.788-0.799)	0.861 (0.850-0.871)
Reinforcement Learning (DDQN)	0.323	0.628	0.788 (0.770-0.806)	0.500 (0.493-0.507)	0.758 (0.745-0.771)
Neural Network	0.442	0.782	0.814 (0.797-0.831)	0.751 (0.746-0.757)	0.874 (0.864-0.884)
Neural Network + SMOTE	0.412	0.756	0.837 (0.821-0.853)	0.683 (0.676-0.689)	0.869 (0.859-0.879)
Neural Network + Cost-Sensitive	**0.470**	**0.792**	0.768 (0.750-0.787)	0.816 (0.811-0.822)	0.872 (0.862-0.882)
XGBoost	0.456	0.791	0.810 (0.793-0.827)	0.773 (0.767-0.779)	0.877 (0.867-0.887)
XGBoost + SMOTE	0.457	**0.790**	0.794 (0.777-0.812)	0.786 (0.780-0.791)	0.876 (0.866-0.886)
XGBoost + Cost-Sensitive	0.434	0.776	0.819 (0.802-0.836)	0.736 (0.730-0.742)	0.869 (0.859-0.879)
**PUH**					
Reinforcement Learning (Q-imb)	0.327	0.754	0.794 (0.776-0.812)	0.716 (0.711-0.720)	0.831 (0.819-0.842)
Reinforcement Learning (DDQN)	0.262	0.661	0.756 (0.737-0.774)	0.578 (0.573-0.584)	0.762 (0.750-0.774)
Neural Network	0.331	0.760	0.825 (0.808-0.842)	0.699 (0.695-0.704)	0.860 (0.849-0.870)
Neural Network + SMOTE	0.320	0.746	0.818 (0.802-0.835)	0.681 (0.676-0.685)	0.853 (0.842-0.863)
Neural Network + Cost-Sensitive	0.332	0.76	0.809 (0.792-0.826)	0.714 (0.709-0.718)	0.850 (0.839-0.861)
XGBoost	**0.363**	**0.792**	0.836 (0.820-0.852)	0.751 (0.746-0.755)	0.881 (0.871-0.891)
XGBoost + SMOTE	0.351	**0.781**	0.834 (0.818-0.851)	0.730 (0.726-0.735)	0.881 (0.871-0.890)
XGBoost + Cost-Sensitive	**0.364**	**0.792**	0.829 (0.813-0.846)	0.757 (0.752-0.761)	0.881 (0.871-0.891)
**UHB**					
Reinforcement Learning (Q-imb)	**0.327**	0.778	0.765 (0.726-0.805)	0.790 (0.782-0.798)	0.837 (0.814-0.861)
Reinforcement Learning (DDQN)	0.214	0.598	0.761 (0.721-0.801)	0.471 (0.461-0.480)	0.721 (0.694-0.749)
Neural Network	0.325	**0.785**	0.820 (0.784-0.856)	0.752 (0.744-0.761)	0.867 (0.846-0.889)
Neural Network + SMOTE	0.302	0.763	0.825 (0.789-0.860)	0.705 (0.696-0.714)	0.848 (0.825-0.871)
Neural Network + Cost-Sensitive	**0.336**	**0.782**	0.756 (0.716-0.796)	0.808 (0.801-0.816)	0.861 (0.839-0.883)
XGBoost	0.321	0.774	0.770 (0.731-0.809)	0.779 (0.770-0.787)	0.861 (0.839-0.883)
XGBoost + SMOTE	0.312	0.763	0.754 (0.714-0.794)	0.773 (0.764-0.781)	0.853 (0.830-0.876)
XGBoost + Cost-Sensitive	0.308	0.764	0.774 (0.735-0.814)	0.753 (0.745-0.762)	0.851 (0.829-0.874)
**BH**					
Reinforcement Learning (Q-imb)	**0.617**	**0.837**	0.799 (0.733-0.864)	0.878 (0.858-0.898)	0.867 (0.829-0.906)
Reinforcement Learning (DDQN)	0.349	0.625	0.660 (0.582-0.737)	0.592 (0.562-0.622)	0.706 (0.656-0.756)
Neural Network	0.589	0.820	0.778 (0.710-0.846)	0.865 (0.845-0.886)	0.885 (0.849-0.921)
Neural Network + SMOTE	0.553	0.801	0.764 (0.695-0.833)	0.840 (0.818-0.863)	0.879 (0.842-0.916)
Neural Network + Cost-Sensitive	**0.614**	0.815	0.736 (0.664-0.808)	0.902 (0.884-0.920)	0.883 (0.847-0.920)
XGBoost	0.575	**0.822**	0.806 (0.741-0.870)	0.838 (0.816-0.861)	0.894 (0.859-0.929)
XGBoost + SMOTE	0.556	0.797	0.743 (0.672-0.814)	0.855 (0.833-0.876)	0.885 (0.849-0.921)
XGBoost + Cost-Sensitive	0.551	0.811	0.812 (0.749-0.876)	0.810 (0.786-0.834)	0.889 (0.854-0.925)

G.3 Multiclass Patient Diagnosis

Table 12: Individual sensitivities per acute event class (alongside 95% CIs), calculated using a "one-vs-all" method.

Label	Cardiovascular	Respiratory	Gastrointestinal	Systemic	Renal
Reinforcement Learning (Q-imb)	0.853 (0.837-0.869)	0.677 (0.657-0.697)	0.897 (0.871-0.923)	0.767 (0.741-0.793)	0.545 (0.508-0.582)
Reinforcement Learning (DDQN)	0.725 (0.704-0.746)	0.593 (0.572-0.615)	0.905 (0.880-0.930)	0.689 (0.661-0.718)	0.440 (0.403-0.478)
Neural Network	0.900 (0.886-0.914)	0.754 (0.735-0.773)	0.895 (0.869-0.921)	0.606 (0.576-0.636)	0.419 (0.382-0.456)
Neural Network + SMOTE	0.901 (0.887-0.915)	0.783 (0.765-0.801)	0.893 (0.866-0.920)	0.592 (0.562-0.622)	0.400 (0.363-0.437)
Neural Network + Cost-Sensitive	0.926 (0.914-0.938)	0.524 (0.502-0.546)	0.905 (0.880-0.930)	0.742 (0.715-0.769)	0.465 (0.428-0.502)
XGBoost	0.864 (0.848-0.880)	0.849 (0.833-0.865)	0.897 (0.871-0.923)	0.633 (0.604-0.662)	0.423 (0.386-0.460)
XGBoost + SMOTE	0.863 (0.847-0.879)	0.853 (0.838-0.868)	0.903 (0.877-0.929)	0.626 (0.597-0.655)	0.422 (0.385-0.459)
XGBoost + Cost-Sensitive	0.861 (0.845-0.877)	0.664 (0.643-0.685)	0.907 (0.882-0.932)	0.774 (0.749-0.799)	0.515 (0.478-0.552)

Table 13: Individual G values per acute event class, calculated using a “one-vs-all” method.

Label	Cardiovascular	Respiratory	Gastrointestinal	Systemic	Renal
Reinforcement Learning (Q-imb)	0.906	0.780	0.938	0.830	0.714
Reinforcement Learning (DDQN)	0.799	0.522	0.931	0.651	0.681
Neural Network	0.889	0.803	0.941	0.754	0.643
Neural Network + SMOTE	0.891	0.812	0.940	0.748	0.630
Neural Network + Cost-Sensitive	0.873	0.703	0.944	0.817	0.669
XGBoost	0.912	0.823	0.942	0.770	0.647
XGBoost + SMOTE	0.912	0.824	0.945	0.767	0.646
XGBoost + Cost-Sensitive	0.911	0.769	0.947	0.830	0.694

G.4 Training Times

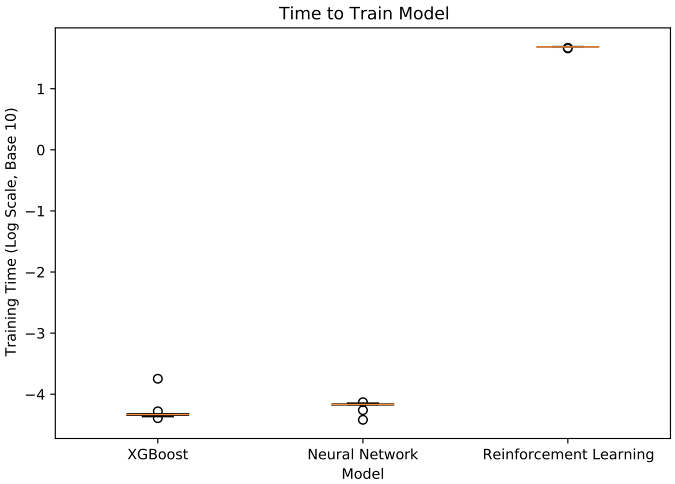

Figure 3: Average training times across 10 training runs, for XGBoost, Neural Network, and Reinforcement Learning Models. Values displayed on logarithmic (base 10) scale.

Although the time taken to train a reinforcement learning-based model increases, there is the advantage of increased flexibility of applying the approach to all types of learning, rather than limiting the performance on tabular data (i.e. because the approach is demonstrated with neural networks, the principles can be generalised to imbalanced image recognition problems, as well as NLP problems, which a model such as XGBoost is not appropriate for).
